# New species and records of the subgenus Libnotes (Laosa) Edwards (Diptera, Limoniidae) from China with a key to world species

**DOI:** 10.3897/zookeys.1041.65906

**Published:** 2021-06-01

**Authors:** Zehui Kang, Xiao Zhang

**Affiliations:** 1 Key Lab of Integrated Crop Pest Management of Shandong Province, College of Plant Health and Medicine, Qingdao Agricultural University, Qingdao 266109, China Qingdao Agricultural University Qingdao China

**Keywords:** Chinese fauna, crane flies, Limoniinae, new record, taxonomy

## Abstract

Twenty species of Libnotes (Laosa) Edwards, 1926 are known worldwide and three are known from China so far. Here, two species of *Laosa* are added to the Chinese fauna, of which L. (L.) baiyunensis**sp. nov.** is described and illustrated as new to science, and L. (L.) fuscinervis Brunetti, 1912 is newly recorded from China. Morphologically, the new species is most similar to L. (L.) charmosyne (Alexander, 1958) but can be distinguished by the pleura of the thorax, the relative position of the additional cross veins in cell r_3_ and r_5_, and the details of the male genitalia. A key to the world species of *Laosa* is presented.

## Introduction

*Libnotes* Westwood, 1876 is a species-rich Limoniidae genus with a total number of 293 species and subspecies, separated into eight subgenera: *Afrolimonia* Alexander, 1965, *Goniodineura* van der Wulp, 1895, *Gressittomyia* Alexander, 1936a, *Laosa* Edwards, 1926, *Libnotes* (s. str.), *Metalibnotes* Alexander, 1972, *Neolibnotes* Alexander, 1972 and Paralibnotes Alexander, 1972. The subgenus Laosa constitutes a small group within the genus with 20 known species from the Oriental (nine species), Australasian/Oceanian (eight species) and Palaearctic (three species) regions (Oosterbroek 2021), and here an additional new species from China is described and illustrated. It can be easily distinguished from other subgenera by the wing having two additional cross veins in cells r_3_ (r-r) and r_5_ (r-m, absent in some species) and Sc_1_ ending far beyond the fork of Rs. Detailed features for recognition were given by Edwards (1926) and Podenas and Byun (2018).

Three species of the subgenus Laosa were previously recorded from China: L. (L.) diphragma (Alexander, 1934a), L. (L.) regalis Edwards, 1916 and L. (L.) transversalis de Meijere, 1916. In this paper, two *Laosa* species are added to the Chinese fauna, of which L. (L.) baiyunensis sp. nov. is described and illustrated as new to science and L. (L.) fuscinervis Brunetti, 1912, known previously only from India, is newly recorded from China. A key to the world species of *Laosa* based on types and non-type specimens, and on the literature is presented.

## Material and methods

Specimens for this study were collected from several localities in China by different entomologists between 2002–2016. Type specimens are deposited in the Entomological Museum of China Agricultural University, Beijing, China (**CAU**). Other studied specimens are deposited in Qingdao Agricultural University, Shandong, China (**QAU**). We also examined specimens from the National Museum of Natural History, Smithsonian Institution, Washington D.C., USA (**USNM**) and the Natural History Museum, London, UK (**NHM**) (Table 1). Genitalic preparations of males were made by macerating the apical portion of the abdomen in cold 10% NaOH for 12–15 hours. Observations and illustrations were made using a ZEISS Stemi 2000-C stereomicroscope. Photographs were taken with a Canon EOS 77D digital camera through a macro lens. Details of coloration were examined in specimens immersed in 75% C_2_H_5_OH.

**Table 1.** Information of the examined specimens from USNM and NHM.

Species	Specimens examined	Collection
L. (L.) charmosyne	Holotype, male, Japan: Shikoku, Mt. Ishizachi (1800 m), 1956.VI.16, T. Yano.	USNM
L. (L.) fuscinervis	Paratype, male, India: East Himalayas, Dajiling (1829 m), 1908.IX.22, E. Brunetti.	NHM
L. (L.) kariyana	Holotype, male, Japan: Honshu, Ontake (1800 m), 1934.VII.6–10, H. Ise.	USNM
L. (L.) manobo	Holotype, male, Philippines: Mindanao, Mt. Apo (1981 m), 1930.IX.14, C. F. Clagg.	USNM
L. (L.) noctipes	Holotype, female, India: Sikkim, karponang (2469 m), 1959.VIII.22, Schmid.	USNM
L. (L.) regalis	Holotype, male?, China: Taiwan, Taihoku, T. Shiraki. Other material: 1 male, China: Taiwan, Arisan, 1917.IV.20, T. Shiraki.	NHM
L. (L.) rotundifolialeos	Paratypes, 2 males 2 females, Indonesia: Sulawesi Utara, Dumoga-Bone National Park (211 m), 1985.VIII. 19–30, Chen W. Young.	NHM
L. (L.) taficola	Holotype, female, Papua New Guinea: Mt Tafa (2591 m), 1934.III, L. E. Cheesman.	NHM
L. (L.) transversalis	1 male, China: Taiwan, Arisan, 1919.IV.25, T. Shiraki.	NHM

The morphological terminology mainly follows McAlpine (1981), and that for venation follows Alexander and Byers (1981). The following abbreviations in figures are used: **tg 9** = ninth tergite, **tg 10** = tenth tergite, **goncx** = gonocoxite, **o gonst** = outer gonostylus, **i gonst** = inner gonostylus, **aed** = aedeagus, **pm** = paramere, **cerc** = cercus, **hyp vlv** = hypogynial valve.

## Taxonomy

### Key to world species of *Laosa*

**Table:** 

1	Basal 1/4 of wing with complete or broken crossband; m-m shorter than basal section of M_3_ (Fig. 1a–c)	**2**
–	Basal 1/4 of wing without conspicuous crossband; m-m significantly longer than basal section of M_3_ (Figs 2d, 4d)	**9**
2 (1)	Wing with broad and complete crossband extending from cord to distal end of cell dm (Fig. 1c)	**3**
–	Wing without broad or complete crossband extending from cord to distal end of cell dm (Fig. 1a, b)	**5**
3 (2)	Tip of wing narrowly falcate	**L. (L.) falcata (Alexander, 1935)**
–	Tip of wing round	**4**
4 (3)	Rs nearly straight or slightly curved, r-r far beyond r-m and distance between them more than twice length of r-r (Fig. 1c)	**L. (L.) rotundifolialeos (Young, 1990)**
–	Rs strongly arcuated, r-r beyond r-m and distance between them about length of r-r	**L. (L.) innuba (Alexander, 1941)**
5 (2)	Crossvein r-r situated before r-m, basal section of CuA_1_ at fork of M (Fig. 1b)	**L. (L.) iris (Alexander, 1950)**
–	Crossvein r-r situated beyond r-m, basal section of CuA_1_ distinctly beyond fork of M (Figs 1a, c, 2d)	**6**
6 (5)	Pleura pale yellow without dark area	**L. (L.) bipartita (Alexander, 1936b)**
–	Pleura with conspicuous dark area	**7**
7 (6)	Basal section of CuA_1_ slightly beyond fork of M and at about 1/8 of cell dm	**L. (L.) manobo (Alexander, 1931)**
–	Basal section of CuA_1_ far beyond fork of M and at 1/4–1/2 of cell dm (Figs 2d, 4d)	**8**
8 (7)	Coxae yellow; R_2_ far before tip of Sc_2_ and distance between them about twice length of R_2_, tip of A_1_ bent very strongly to wing margin	**L. (L.) pavo (Alexander, 1964)**
–	Coxae brown; R_2_ before tip of Sc_2_ and distance between them about length of R_2_, tip of A_1_ slightly curved	**L. (L.) suffalcata (Alexander, 1964)**
9 (1)	Wing without additional cross vein in cell r_5_ (Fig. 4d)	**10**
–	Wing with additional cross vein in cell r_5_ (Figs 1a–c, 2d)	**15**
10 (9)	Wing with stripes along veins broad and extensive, nearly covering wing tip	**L. (L.) noctipes (Alexander, 1967)**
–	Wing with stripes along veins not as broad or extensive	**11**
11 (10)	Crossvein m-m about four times or more as long as basal section of M_3_	**12**
–	Crossvein m-m less than three times as long as basal section of M_3_ (Fig. 4d)	**13**
12 (11)	Wing with many conspicuous spots; R_2_ and r-r distinct before distal end of cell dm	**L. (L.) taficola (Alexander, 1948)**
–	Wing nearly unpatterned except very light brown spots at fork of Sc and over tip of Sc_2_; R_2_ distinct beyond distal end of cell dm, r-r aligned with distal end of cell dm	**L. (L.) transversalis de Meijere, 1916**
13 (11)	Anterior scutum and pleura dark brown, without conspicuous pattern	**L. (L.) dolonigra (Alexander, 1956)**
–	Anterior scutum and pleura with conspicuous stripes (Fig. 4c)	**14**
14 (13)	Body length of male more than 13.0 mm; r-r aligned with distal end of cell dm (Alexander 1967)	**L. (L.) impensa (Alexander, 1967)**
–	Body length of male less than 10.0 mm; r-r distinctly before distal end of cell dm (Fig. 4d)	**L. (L.) fuscinervis Brunetti, 1912**
15 (9)	R_2_ far before tip of Sc_2_	**16**
–	R_2_ close to tip of Sc_2_ (Fig. 2d)	**17**
16 (15)	Antennal scape yellow, pedicel and flagellomeres dark brown; anterior scutum with four yellow stripes; Sc relatively short, end aligned with base of cell dm (Alexander 1959)	**L. (L.) joculator (Alexander, 1959)**
–	Antenna black throughout; anterior scutum with three confluent dark brown stripes; Sc long, end aligned with middle of cell dm	**L. (L.) kariyana (Alexander, 1947)**
17 (15)	Crossvein r-r close to R_2_	**18**
–	Crossvein r-r far before R_2_ and distance between them about or more than length of r-r (Figs 2d, 4d)	**19**
18 (17)	Anterior scutum with indistinct median stripe; wing length of male 10.0–15.0 mm, r-m distinctly before distal end of cell dm, tip of A_2_ nearly straight or slightly curved	**L. (L.) charmosyne (Alexander, 1958)**
–	Anterior scutum with four ill-defined stripes; wing length of male about 25.0 mm, r-m aligned with distal end of cell dm, tip of A_2_ bent very strongly toward margin	**L. (L.) regalis Edwards, 1916**
19 (17)	Axillary region of wing without spots (Fig. 2d)	**L. (L.) baiyunensis sp. nov.**
–	Axillary region of wing darkened (Fig. 4d)	**20**
20 (19)	Tibiae yellow with broad, brown subbasal rings; r-r far before R_2_ and distance between them about 1.5 times length of r-r	**L. (L.) riedelella (Alexander, 1934b)**
–	Tibiae brownish yellow without subbasal ring; r-r before R_2_ and distance between them less than length of r-r	**L. (L.) diphragma (Alexander, 1934a)**

**Figure 1.**
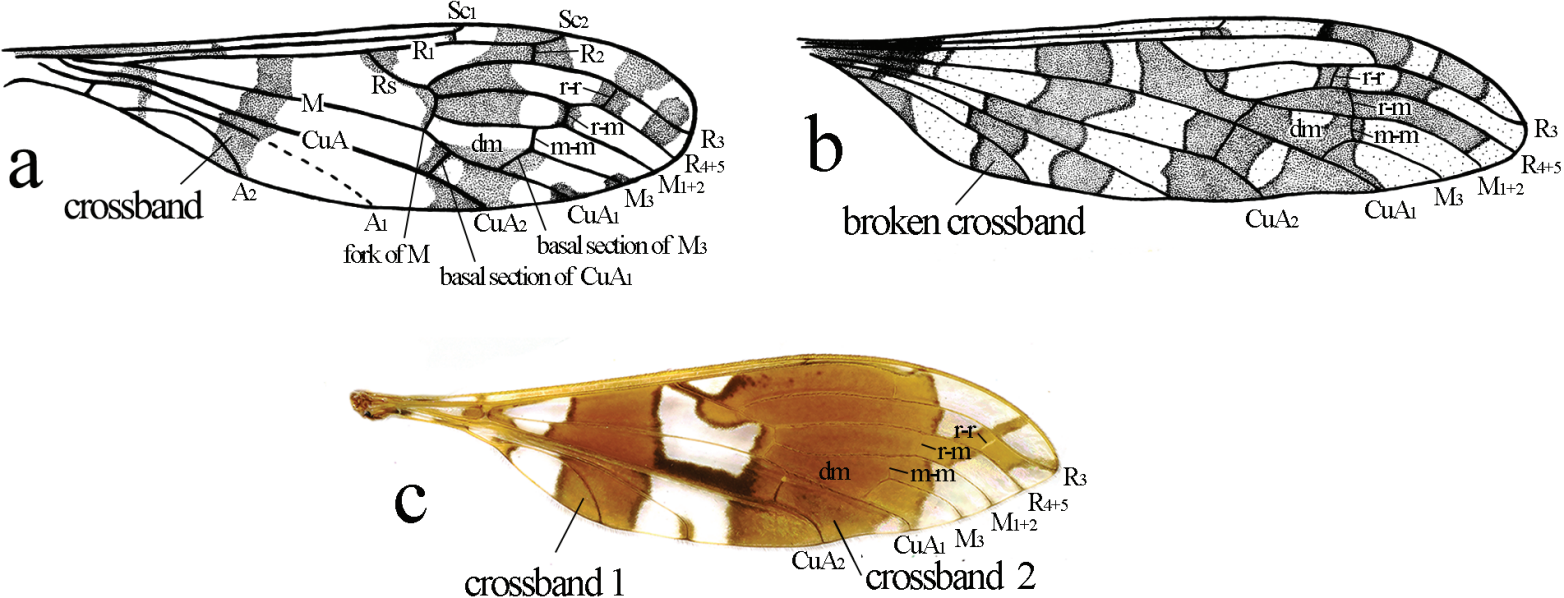
Wings of Libnotes (Laosa)**a**L. (L.) bipartita (from Alexander 1936b) **b**L. (L.) iris (form Edwards 1926) **c**L. (L.) rotundifolialeos (paratype, photo by Jinlong Ren).

#### Libnotes (Laosa) baiyunensis sp. nov.

Taxon classificationAnimaliaDipteraLimoniidae

6395D881-A8E6-5CC8-B02C-4FC3A3C84288

http://zoobank.org/EE267965-E009-46B7-B13D-6129B6F06E2A

Figs 2 , 3

##### Specimens examined.

***Holotype*,** male (CAU), China: Henan, Songxian, Mt. Baiyun, 2002.VII.22, Ding Yang. ***Paratypes***: 1 male (CAU), same data as holotype. 1 male 1 female (CAU), China: Henan, Songxian, Mt. Baiyun (1500 m), 2008.VIII.14, Ding Yang.

##### Diagnosis.

Anterior scutum brown with side edges brownish black. Pleura brownish yellow with a broad brownish black stripe extending from cervical region to base of wing. Tip of wing round. Wing nearly unpatterned except some pale brown patches around cross veins and portions of longitudinal veins, without conspicuous crossband from top to bottom. Sc long, ending near middle of cell dm. Rs slightly curved. R_2_ slightly before tip of Sc_2_. Two additional cross veins in cells r_3_ and r_5_, the former (r-r) beyond distal end of cell dm, the latter (r-m) aligned with distal end of cell dm; m-m twice as long as basal section of M_3_. Basal section of CuA_1_ far beyond fork of M and at about 1/3 of cell dm. Tip of A_2_ nearly straight.

##### Description.

**Male.** Body length 12.0–14.0 mm, wing length 19.0–22.0 mm.

**Figure 2.**
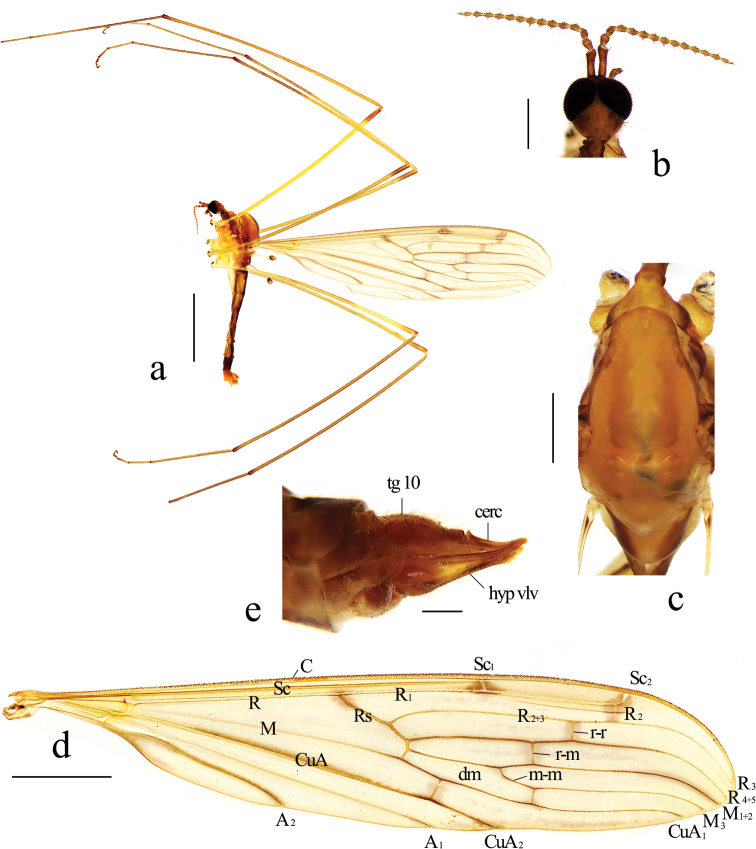
Libnotes (Laosa) baiyunensis sp. nov. **a** habitus of male, lateral view **b** head, dorsal view **c** thorax, dorsal view **d** wing **e** female ovipositor, lateral view. Scale bars: 5.0 mm (**a**); 3.0 mm (**d**); 1.0 mm (**b, c**); 0.2 mm (**e**).

***Head*** (Fig. 2b). Brown. Hairs on head brown. Antenna length 2.9 mm, brown. Scape long cylindrical; pedicel oval, nearly as long as first flagellomere; flagellomeres oval, tapering apically, terminal flagellomere 1.5 times as long as preceding segment. Mouthparts brown with white hairs; palpus brown with brown hairs.

***Thorax*** (Fig. 2c). Pronotum brown with sides brownish black. Prescutum brown with side edges brownish black. Anterior scutum brown with side edges brownish black; posterior scutum brown. Scutellum pale brown. Mediotergite pale brown with sides brownish black. Pleura (Fig. 2a) brownish yellow with a broad brownish black stripe extending from cervical region to base of wing. Hairs on thorax white. Coxae yellow; trochanters pale yellow; femora yellow to brownish yellow with tips dark brown; tibiae brown; tarsi brown. Hairs on legs dark brown. Wing (Fig. 2d) tinged with pale brownish yellow. Darkened areas around cross veins, distal end of cell dm and CuA_1_, tip of M_1+2_, CuA and A_2_; three small spots at base of Rs, at fork of Sc, and over R_2_ and tip of Sc_2_. Venation: Sc long, ending far beyond fork of Rs and near middle of cell dm. Basal section of Sc_2_ very close to tip of Sc_1_. Tip of Sc_2_ nearly transverse, indistinct at wing margin. Rs very short, slightly sinuous. R_2_ slightly before tip of Sc_2_. Radial and medial veins distinctly curved caudally before wing margin. Two additional cross veins in cells r_3_ and r_5_, the former (r-r) at middle of cell r_3_, the latter (r-m) at basal 2/5 of cell r_5_ and aligned with distal end of cell dm. Cell dm elongate, more than 5 times as long as its width; m-m elongate, twice as long as basal section of M_3_. Basal section of CuA_1_ far beyond fork of M and at about 1/3 of cell dm. A_1_ straight. A_2_ slightly sinuous. Halter length 2.6 mm, yellow with knob brown.

***Abdomen*.** Tergites brownish yellow with a brown median stripe, lateral borders brown; eighth tergite brown. Sternites brownish yellow with eighth sternite brown. Hairs on abdomen white.

**Figure 3.**
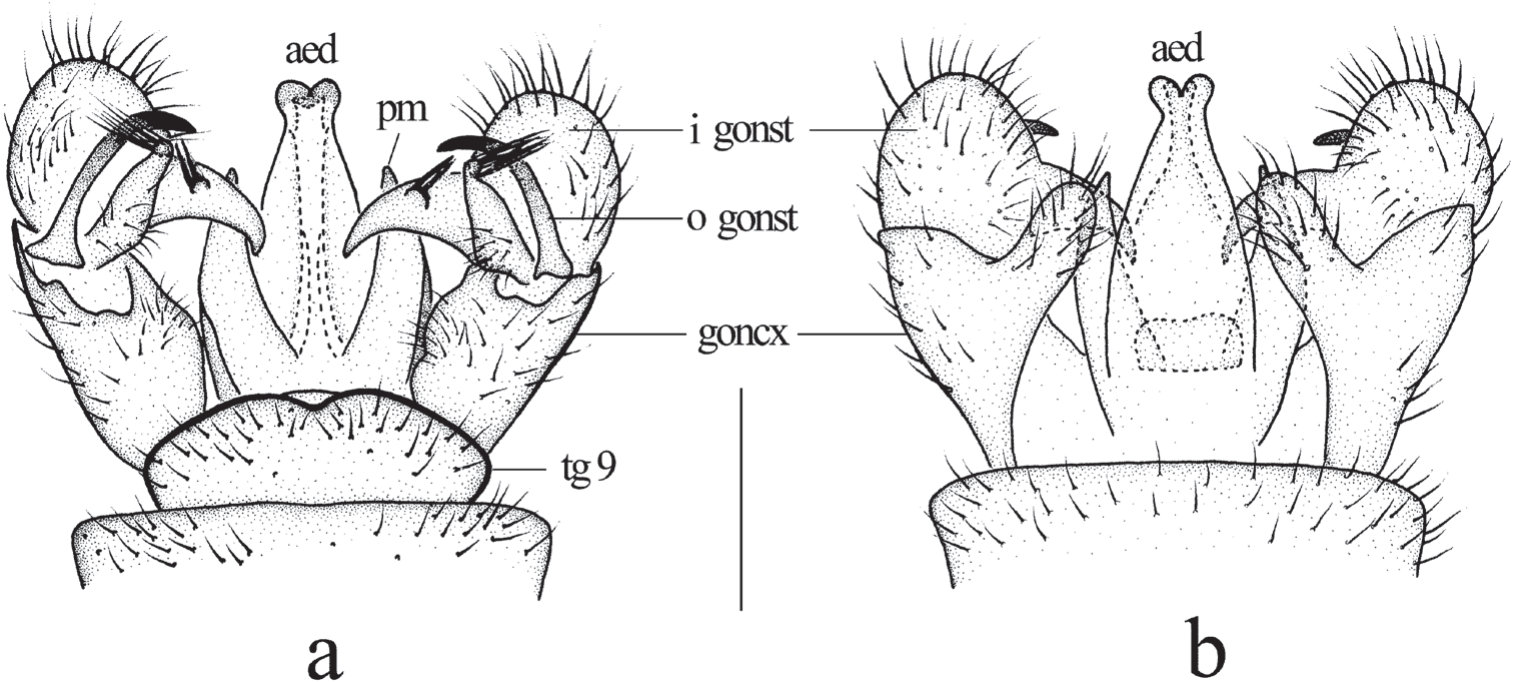
Libnotes (Laosa) baiyunensis sp. nov. **a** male hypopygium, dorsal view **b** male hypopygium, ventral view. Scale bars: 0.5 mm.

***Hypopygium*** (Fig. 3). Ninth tergite with widely rounded posterior margin and small median emargination. Gonocoxite elongate, slender with an elongate, blunt-apexed ventromesal lobe; inside edge with small setose bulge. Outer gonostylus arched at 2/3 length, tip acute. Inner gonostylus short, oval with long, arched rostral prolongation armed with two spines near base from a single tubercle; an elongate lobe arising dorsally near base with a brush of long setae at apex, at right angle to lobe and directed laterally. Paramere wide at base, elongate, triangular distally. Penis long, tip sunken in the middle.

**Female.** Body length 11.5 mm, wing length 17.5 mm. Similar to male, but eighth tergite brownish yellow with a broad brown median stripe. Tenth tergite brown. Cercus (Fig. 2e) brown, tip slightly exceeding tip of hypogynial valve. Hypogynial valve brownish yellow with borders darker, base slightly beyond base of tenth tergite.

##### Etymology.

The species is named after the type locality Mt. Baiyun.

##### Distribution.

China (Henan).

##### Remarks.

This species is somewhat similar to L. (L.) charmosyne from South Korea and Japan in having similar spots on the wing, but it can be easily distinguished from the latter by the pleura of the thorax being brownish yellow with a broad brownish black stripe, the crossvein r-r being 1.5 to 2 times its length before R_2_, the crossvein r-m being aligned with the distal end of cell dm, the basal section of CuA_1_ being far beyond the fork of M and at about 1/3 of cell dm (Fig. 2d), and the inner gonostylus being about 2/3 length of the gonocoxite (Fig. 3a), whereas in L. (L.) charmosyne, the pleura of the thorax is dark brownish gray, the crossvein r-r is near R_2_, the crossvein r-m is distinctly before the distal end of cell dm, the basal section of CuA_1_ is beyond the fork of M and at 1/6–1/5 of cell dm, and the inner gonostylus is half the length of the gonocoxite (Alexander 1958; Podenas and Byun 2018).

#### Libnotes (Laosa) fuscinervis

Taxon classificationAnimaliaDipteraLimoniidae

Brunetti, 1912

048C847A-35E8-58A0-B3D2-CFFD6E2664D5

Figs 4 , 5

Libnotes fuscinervis Brunetti, 1912: 411. Type locality: Dajiling, East Himalayas (India).

##### Specimens examined.

***Paratype*,** male (NHM), India: East Himalayas, Dajiling (1829 m), 1908.IX.22, E. Brunetti. **Other material**: 1 male (QAU), China: Yunnan, Lvchun, Yakou (1931 m), 2016.VII.7, Qilemoge.

##### Diagnosis.

Anterior scutum brown with a broad, posteriorly subdivided, dark brown median stripe and a spot on each side of it; posterior half of median stripe with a paler division that broadens out across posterior scutum and scutellum. Pleura brownish yellow with a broad, anteriorly indistinct, brownish black stripe extending from cervical region to mediotergite. Tip of wing round. Wing with many conspicuous spots but without conspicuous crossband from top to bottom. Sc long, ending at 1/3 of cell dm. Rs slightly curved. R_2_ before tip of Sc_2_ and distance between them about length of R_2_. Crossvein r-r before distal end of cell dm. Additional cross vein in cell r_5_ absent; m-m twice as long as basal section of M_3_. Basal section of CuA_1_ far beyond fork of M and at about 1/4 of cell dm. Tip of A_2_ slightly curved.

##### Description.

**Male.** Body length 9.5 mm, wing length 14.5 mm.

**Figure 4.**
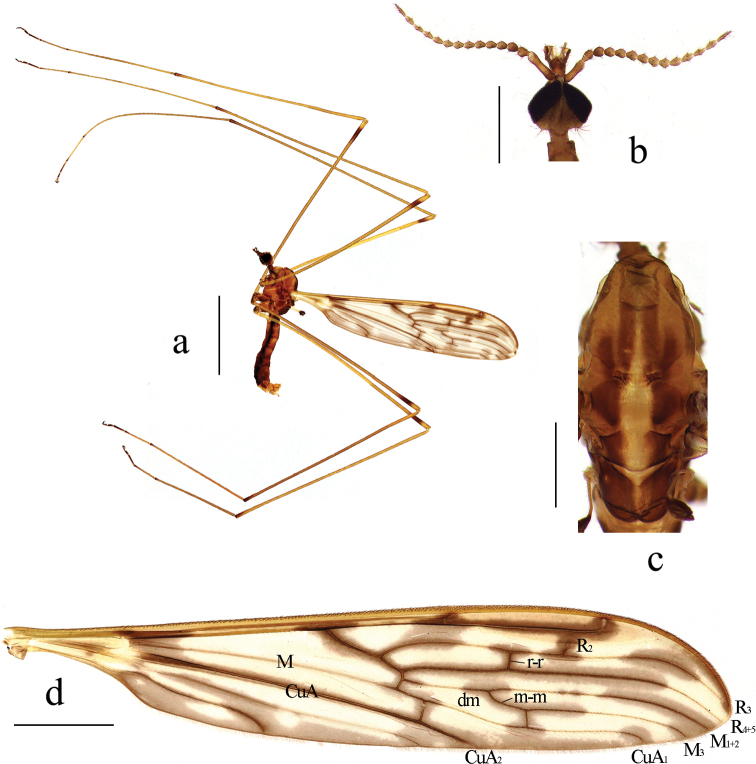
Libnotes (Laosa) fuscinervis Brunetti, 1912 **a** habitus of male, lateral view **b** head, dorsal view **c** thorax, dorsal view **d** wing. Scale bars: 5.0 mm (**a**); 2.0 mm (**d**); 1.0 mm (**b, c**).

***Head*** (Fig. 4b). Brownish yellow. Hairs on head brown. Antenna length 2.0 mm, dark brownish yellow. Scape long cylindrical; pedicel oval, nearly as long as first flagellomere; flagellomeres oval, tapering apically, terminal flagellomere 1.5 times as long as preceding segment. Mouthparts brown with white hairs; palpus brown with brown hairs.

***Thorax*** (Fig. 4c). Pronotum brownish yellow. Prescutum brown. Anterior scutum brown with a broad, posteriorly subdivided, dark brown median stripe and a spot on each side of it; posterior scutum brownish black with a broad yellow median stripe. Scutellum brownish black with a broad yellow median stripe. Mediotergite brownish black with a narrow yellow median stripe. Pleura (Fig. 4a) brownish yellow with a broad, anteriorly indistinct, brownish black stripe extending from cervical region to mediotergite. Hairs on thorax white. Coxae brownish yellow; trochanters yellow; femora brownish yellow with subtips brownish black; tibiae brownish yellow with tips narrowly brownish black; tarsi brownish yellow with tips brownish black. Hairs on legs dark brown. Wing (Fig. 4d) tinged with pale brownish yellow. Many dark patches around cross veins and portions of longitudinal veins as well as patches in cells as shown in Fig. 4d; four darker spots at base of wing, at base of Rs, at fork of Sc, and over R_2_ and tip of Sc_2_, the latter two spots connected by a narrow stripe along Sc_2_. Venation: Sc long, ending far beyond fork of Rs and at 1/3 of cell dm. Basal section of Sc_2_ near tip of Sc_1_. Tip of Sc_2_ nearly transverse, indistinct at wing margin. Rs very short, slightly sinuous. R_2_ before tip of Sc_2_ and distance between them about length of R_2_. Radial and medial veins distinctly curved caudally before wing margin. Crossvein r-r at basal 1/3 of cell r_3_. Cell dm elongate, more than 5 times as long as its width; m-m elongate, twice as long as basal section of M_3_. Basal section of CuA_1_ far beyond fork of M and at about 1/4 of cell dm. A_1_ straight, slightly curved near tip. A_2_ slightly sinuous. Halter length 1.5 mm, pale yellow with knob brownish black.

***Abdomen*.** Tergites brownish yellow with lateral borders brownish black. Sternites brownish yellow, middle of first sternite paler. Hairs on abdomen white.

**Figure 5.**
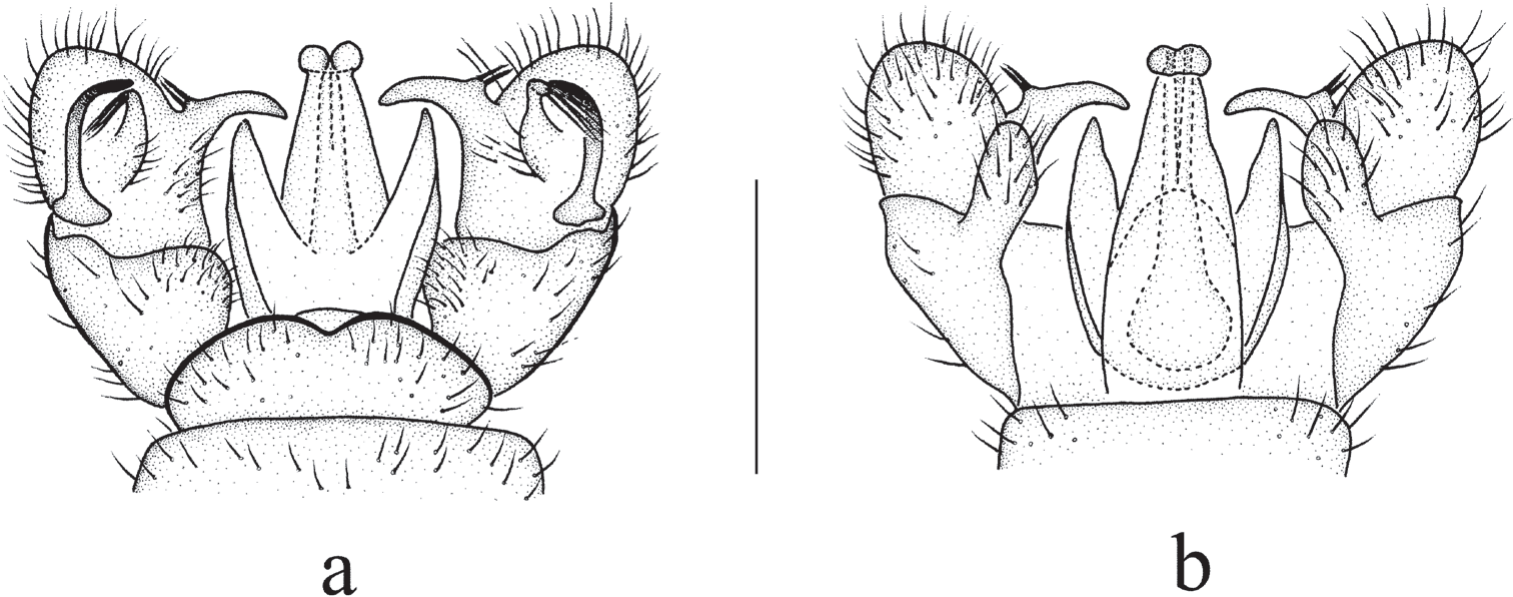
Libnotes (Laosa) fuscinervis Brunetti, 1912 **a** male hypopygium, dorsal view **b** male hypopygium, ventral view. Scale bars: 0.5 mm.

***Hypopygium*** (Fig. 5). Ninth tergite with rounded posterior margin and small median emargination. Gonocoxite stubby with an elongate, blunt-apexed ventromesal lobe; inside edge with a large setose bulge. Outer gonostylus arched at 2/3 length, tip acute. Inner gonostylus short, oval with a long arched rostral prolongation armed with two spines at base from a single tubercle; an elongate lobe arising dorsally near base with a brush of long setae at apex, at right angle to lobe and directed laterally. Paramere wide at base, elongate, triangular distally. Penis long, tip sunken in the middle.

**Female.** Unknown.

##### Distribution.

China (Yunnan); India.

##### Remarks.

This species was known previously only from India. Now it is recorded from China for the first time.

## Supplementary Material

XML Treatment for Libnotes (Laosa) baiyunensis

XML Treatment for Libnotes (Laosa) fuscinervis

## References

[B1] Alexander CP (1931) New or little-known Tipulidae from the Philippines (Diptera). XII. Philippine Journal of Science 46: 447–477.

[B2] Alexander CP (1934a) New or little-known Tipulidae from eastern Asia (Diptera). XIX. Philippine Journal of Science 54: 309–342.

[B3] Alexander CP (1934b) New or little-known Tipulidae from eastern Asia (Diptera). XX. Philippine Journal of Science 54: 433–471.

[B4] Alexander CP (1935) The Diptera of the Territory of New Guinea. II. Family Tipulidae. Proceedings of the Linnaean Society of New South Wales 60: 51–70.

[B5] Alexander CP (1936a) New or little-known Tipulidae from eastern Asia (Diptera). XXXII. Philippine Journal of Science 61: 113–149.

[B6] Alexander CP (1936b) Tipulidae. In: Curran CH (Ed.) The Templeton Crocker expedition to western Polynesian and Melanesian islands, 1933.No. 30. Diptera. Proceedings of the California Academy of Sciences 22(4), 2–11.

[B7] Alexander CP (1941) The Diptera of the Territory of New Guinea. XII. Family Tipulidae. Part IV. Proceedings of the Linnaean Society of New South Wales 66: 138–144.

[B8] Alexander CP (1947) Undescribed species of Japanese crane-flies (Diptera: Tipulidae). Part VI. Annals of the Entomological Society of America 40: 350–371. 10.1093/aesa/40.2.350

[B9] Alexander CP (1948) New or little-known Tipulidae (Diptera). LXXIX. Oriental-Australasian species. Annals and Magazine of Natural History 14(11): 388–414. 10.1080/00222934708654649

[B10] Alexander CP (1950) Notes on the tropical American species of Tipulidae (Diptera). VI. The tribe Limoniini, genus *Limonia*: subgenera *Limonia*, *Neolimnobia*, *Discobola*, and *Rhipidia*. Revista de Entomologia 21: 161–221.

[B11] Alexander CP (1956) New or little-known Tipulidae (Diptera). XCIX. Oriental-Australasian species. Annals and Magazine of Natural History 8(12): 657–674. 10.1080/00222935508655682

[B12] Alexander CP (1958) Undescribed species of Japanese Tipulidae (Diptera). Part I. Transactions of the Shikoku Entomological Society 6: 1–8.

[B13] Alexander CP (1959) New or little-known Tipulidae (Diptera). CVI. Oriental-Australasian species. Annals and Magazine of Natural History 1(13): 657–676. 10.1080/00222935808650994

[B14] Alexander CP (1964) New or little-known Tipulidae from eastern Asia (Diptera). LII. Philippine Journal of Science 92: 383–419.

[B15] Alexander CP (1965) New or little-known Tipulidae from Madagascar (Diptera). Transactions of the American Entomological Society 91: 39–83.

[B16] Alexander CP (1967) New or little-known Tipulidae from eastern Asia (Diptera). LIX. Philippine Journal of Science 95: 79–120.

[B17] Alexander CP (1972) Diptera: Tipulidae. Insects of Micronesia 12: 733–863.

[B18] Alexander CP Byers GW (1981) Tipulidae. In: McAlpine JF Peterson BV Shewell GE Teskey HJ Vockeroth JR Wood DM (Eds) Manual of Nearctic Diptera.Vol. I. Biosystematic Research Centre, Ottawa, 153–190.

[B19] Brunetti E (1912) DipteraNematocera (excluding Chironomidae and Culicidae). Fauna of British India, including Ceylon and Burma 1: 1–581. 10.5962/bhl.title.8711

[B20] Edwards FW (1916) New and little-known Tipulidae, chiefly from Formosa. Annals and Magazine of Natural History 18(8): 245–269. 10.1080/00222931608693846

[B21] Edwards FW (1926) On some crane-flies from French Indo-China. Encyclopedie Entomologique, (B II), Diptera 3: 48–55.

[B22] McAlpine JF (1981) Morphology and terminology, Adults. In: McAlpine JF Peterson BV Shewell GE Teskey HJ Vockeroth JR Wood DM (Eds) Manual of Nearctic Diptera.Vol. I. Biosystematic Research Centre, Ottawa, 9–63.

[B23] Meijere JCH de (1916) Studien uber Sudostasiatische Dipteren, 11. Zur Biologie einiger javanischen Dipteren nebst Beschreibung einiger neuen javanischen Arten. Tijdschrift voor Entomologie 59: 184–213.

[B24] Oosterbroek P (2021) Catalogue of the Craneflies of the World (Diptera, Tipuloidea: Pediciidae, Limoniidae, Cylindrotomidae, Tipulidae). http://ccw.naturalis.nl/ [accessed 10 March 2021]

[B25] Podenas S Byun HW (2018) *Libnotes* crane flies (Diptera: Limoniidae) from Jeju Island (South Korea). Zootaxa 4483: 375–384. 10.11646/zootaxa.4483.2.9 30313794

[B26] Ribeiro GC (2006) Homology of the gonostylus in crane flies, with emphasis on the families Tipulidae and Limoniidae (Diptera, Tipulomorpha). Zootaxa 1110: 47–57. 10.11646/zootaxa.1110.1.5

[B27] Westwood JO (1876) Notae Dipterologicae. No. 2. – Descriptions of some new exotic species of Tipulidae. Transactions of the Entomological Society of London 1876: 501–506. 10.1111/j.1365-2311.1876.tb01926.x

[B28] Wulp FM van der (1895) Eenige Javaansche Diptera. Tijdschrift voor Entomologie 38: 35–48.

[B29] Young CW (1990) A new Limonia (subgenus Laosa) species from Sulawesi (Dipt., Tipulidae) and its resting behaviour. Entomologists Monthly Magazine 126: 239–244.

